# Exogenous Ketone Supplementation Decreased the Lipopolysaccharide-Induced Increase in Absence Epileptic Activity in Wistar Albino Glaxo Rijswijk Rats

**DOI:** 10.3389/fnmol.2019.00045

**Published:** 2019-02-28

**Authors:** Zsolt Kovács, Dominic P. D'Agostino, David M. Diamond, Csilla Ari

**Affiliations:** ^1^Department of Biology, ELTE Eötvös Loránd University, Savaria University Centre, Szombathely, Hungary; ^2^Laboratory of Metabolic Medicine, Department of Molecular Pharmacology and Physiology, Morsani College of Medicine, University of South Florida, Tampa, FL, United States; ^3^Institute for Human and Machine Cognition, Ocala, FL, United States; ^4^Comparative Neuroscience Research Laboratory, Department of Psychology, University of South Florida, Tampa, FL, United States

**Keywords:** ketone supplements, ketosis, LPS, inflammation, absence epilepsy, WAG/Rij rats

## Abstract

It has been demonstrated previously that exogenous ketone supplements such as ketone ester (KE) decreased absence epileptic activity in a well-studied animal model of human absence epilepsy, Wistar Albino Glaxo/Rijswijk (WAG/Rij) rats. It is known that lipopolysaccharide (LPS)-generated changes in inflammatory processes increase absence epileptic activity, while previous studies show that ketone supplement-evoked ketosis can modulate inflammatory processes. Thus, we investigated in the present study whether administration of exogenous ketone supplements, which were mixed with standard rodent chow (containing 10% KE + 10% ketone salt/KS, % by weight, KEKS) for 10 days, can modulate the LPS-evoked changes in absence epileptic activity in WAG/Rij rats. At first, KEKS food alone was administered and changes in spike-wave discharge (SWD) number, SWD time, discharge frequency within SWDs, blood glucose, and beta-hydroxybutyrate (βHB) levels, as well as body weight and sleep-waking stages were measured. In a separate experiment, intraperitoneal (i.p.) injection of LPS (50 μg/kg) alone and a cyclooxygenase 1 and 2 (COX-1 and COX-2) inhibitor indomethacin (10 mg/kg) alone, as well as combined IP injection of indomethacin with LPS (indomethacin + LPS) were applied in WAG/Rij rats to elucidate their influences on SWD number. In order to determine whether KEKS food can modify the LPS-evoked changes in SWD number, KEKS food in combination with IP LPS (50 μg/kg) (KEKS + LPS), as well as KEKS food with IP indomethacin (10 mg/kg) and LPS (50 μg/kg) (KEKS + indomethacin + LPS) were also administered. We demonstrated that KEKS food significantly increased blood βHB levels and decreased not only the spontaneously generated absence epileptic activity (SWD number), but also the LPS-evoked increase in SWD number in WAG/Rij rats. Our results suggest that administration of exogenous ketone supplements (ketogenic foods) may be a promising therapeutic tool in the treatment of epilepsy.

## Introduction

Ketone bodies (beta-hydroxybutyrate/βHB, acetoacetate, and acetone) are produced mainly in the liver, which may serve as an energy source for different tissues under circumstances when glucose supply is insufficient, such as fasting or after following a ketogenic diet (Hashim and VanItallie, 2014; Achanta and Rae, 2017). It has been demonstrated that administration of exogenous ketone (ketogenic) supplements, such as ketone ester (KE) and ketone salt (KS) generate rapid and sustained increases in the blood level of βHB inducing nutritional ketosis (Poff et al., 2015; Ari et al., 2016; Kesl et al., 2016), which may evoke alleviating effects on different central nervous system (CNS) diseases. For example, exogenous ketone supplement-evoked ketosis shows therapeutic potential in the treatment of epilepsy, Parkinson's disease and Alzheimer's disease (D'Agostino et al., 2013; Hashim and VanItallie, 2014; Newport et al., 2015; Kovács et al., 2017).

Inflammatory processes have a role in the precipitation and aggravation of epileptic activity (Vezzani et al., 2008, 2011). For example, it was demonstrated in a well-investigated animal model of human absence epilepsy of Wistar Albino Glaxo/Rijswijk (WAG/Rij) rats (Kovács et al., 2006, 2011, 2014a; Russo et al., 2014) that lipopolysaccharide (LPS; a cell wall component of gram-negative bacteria) (Vezzani et al., 2011) aggravates absence epileptic activity. LPS increases the expression of Toll-like receptor 4 (TLR4) and the release/expression of proinflammatory cytokines, such as interleukin-1β (IL-1β), cyclooxygenase-2 (COX-2) and, as a consequence, prostaglandins (Mlodzikowska-Albrecht et al., 2007; Vezzani et al., 2011). Prostaglandins may decrease the seizure threshold and prostaglandin E_2_ (PGE2) showed a proconvulsant effect (Sayyah et al., 2003). These effects of LPS may enhance cortical excitability for example via modulation of GABAergic, glutamatergic, and adenosinergic systems (Wang and White, 1999; Coenen and Van Luijtelaar, 2003; Kovács et al., 2015b) and, as a consequence, augment absence epileptic activity in WAG/Rij rats (Kovács et al., 2006, 2011, 2014a). The LPS-evoked increase in spike-wave discharge (SWD) number was abolished by a potent inhibitor of prostaglandin synthesis, a cyclooxygenase 1 (COX-1) and COX-2 inhibitor indomethacin. These results suggest the role of the TLR4/COX-2/PGE2 system in the LPS-induced processes leading to PGE2 synthesis, which may enhance SWD generator mechanisms (Kovács et al., 2006, 2011, 2014a).

It has been demonstrated that intragastric administration (gavage) of ketone supplements, such as KE, decreased absence epileptic activity (SWDs) in WAG/Rij rats (Kovács et al., 2017). The exogenous ketone supplement-evoked increase in βHB level may exert its alleviating effects on CNS diseases, among others, via modulation of the inflammatory system (Newman and Verdin, 2014; Youm et al., 2015; Yamanashi et al., 2017), which is implicated in the pathophysiology of absence epilepsy (Kovács et al., 2006, 2011; Van Luijtelaar et al., 2012; Russo et al., 2014). Consequently, we hypothesized that administration of exogenous ketone supplements may modulate not only absence epileptic activity, which spontaneously generated and manifested in SWDs as shown by an electroencephalogram (EEG) (Coenen and Van Luijtelaar, 2003; Kovács et al., 2017), but also the LPS-induced changes in absence epileptic activity in WAG/Rij rats (Kovács et al., 2006, 2014a). Thus, we addressed in the present study whether sub-chronical administration (10 days) of exogenous ketone supplements, which were mixed with standard rodent chow (10% KE + 10% KS, % by weight: KEKS), can modulate spontaneous absence epileptic activity and LPS-evoked increases in SWD number.

## Materials and Methods

### Animals

Animal treatments and surgery procedures were carried out according to the local ethical rules, guidelines of the Hungarian Act of Animal Care and Experimentation (1998, XXVIII, section 243), European Communities Council Directive 24 November 1986 (86/609/EEC) and EU Directive 2010/63/EU on the use and treatment of animals in experimental laboratories. The experiments were approved by the Animal Care and Experimentation Committee of the Eötvös Loránd University (Savaria Campus) and National Scientific Ethical Committee on Animal Experimentation (Hungary) under license number VA/ÉBNTF02/85-8/2016. All efforts were made to minimize pain and suffering and to reduce the number of animals used.

WAG/Rij male rats (*n* = 48; 10 months old, 325–345 g; breeding colony of WAG/Rij rats at Eötvös Loránd University, Savaria Campus, Szombathely, Hungary) were housed in groups (3–4 animals/group), while they were housed individually after surgery. Standard laboratory conditions were provided during the experiments (12:12 h light-dark cycle: light was on from 8:00 a.m. to 8:00 p.m.; free access to water and food; air-conditioned rooms at 22 ± 2°C).

### Electrode Implantation and EEG Recording

Isoflurane-air mixture (2.0–2.5%) anesthesia was used for electrode implantation for EEG recording (Kovács et al., 2006). Briefly, screw electrodes were implanted into the bone above two cortical areas (primary motor cortex and somatosensory cortex: A 0.8 mm, L 1.8 mm, and A 0.2 mm, L 6.2 mm, respectively) (Paxinos and Watson, 1999) and above the cerebellar cortex (as ground electrode). The reference electrode was a stainless steel plate (3 × 4 mm with one side insulated), which was implanted under the skin and over the masseter muscle. All electrodes and the plate were soldered to a ten-pin socket, which were fixed and attached to the skull by dentacrylate cement (Ivoclar, Liechtenstein). Lidocaine ointment (5%; EGIS, Hungary) was used for post-operative pain relief (Kovács et al., 2006).

Electroencephalography was recorded by a differential biological amplifier (Bioamp4, Supertech Ltd., Pécs, Hungary) attached to a CED 1401 mkII (Cambridge Electronic Design Ltd., UK) data capture and analysis device between 2:30 p.m. and 5:00 p.m. The bandwidth of the EEG recording was 0.3–150 Hz and the sampling rate was 500 Hz (Kovács et al., 2014a).

### Administration of Ketone Supplements and Drugs

Both KE (R,S-1,3-butanediol—acetoacetate diester) and KS (Na^+^/K^+^-βHB mineral salt) were developed by D'Agostino et al. (2013; University of South Florida/USF, USA) in collaboration with Savind Inc. (Urbana, IL, USA). Ketone salt was mixed into a 50% solution (375 mg/g pure βHB and 125 mg/g of Na^+^/K^+^ in a 1:1 ratio). As we tested in a pilot study, 20% KEKS in standard rodent chow (KEKS food) was well-palatable for rats without either side effects or decrease in body weight and was able to induce ketosis (10% KE + 10% KS, % by weight; mixed with powdered standard rodent chow and water resulting paste-like consistency food). In order to improve palatability 1% saccharine was added. Moreover, *ad libitum* feeding of rats by ketone-supplemented food is a non-stressful method for ketone supplement-induced ketosis. Thus, in this study we fed the animals with KEKS-containing (20% KEKS) food for 10 days. To ensure freshness and *ad libitum* access, KEKS food was freshly mixed and replaced every day and was placed in a Petri dish on the bottom of the animal's cage.

Based on our previous results on WAG/Rij rats (Kovács et al., 2006, 2014a), the effective doses of LPS (50 μg/kg; Sigma-Aldrich Inc., Hungary, Budapest) and indomethacin (10 mg/kg; Sigma-Aldrich Inc., Hungary, Budapest) were also IP injected alone and in combination. It was demonstrated earlier that 5% (v/v) ethanol solution has no significant effect on absence epileptic activity in WAG/Rij rats (Kovács et al., 2006, 2014a). Thus, 5% ethanol solution was administered to dissolve the indomethacin, whereas LPS was dissolved in saline (Kovács et al., 2006, 2014a).

### Experimental Design

#### Adaptation Period and Pre-treatment Control Days

**Figure 1:**
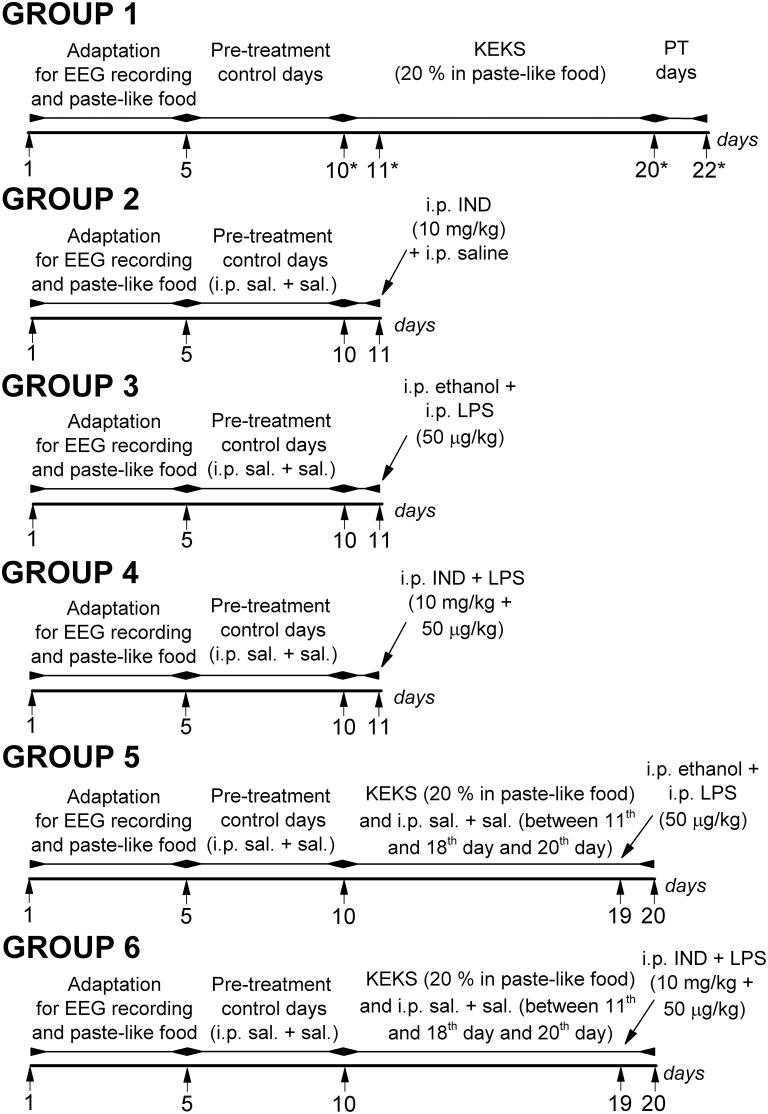
Schematic drawing of the experimental design on 6 animal groups. *Days of blood glucose and βHB level measuring (group 1); IND, indomethacin; i.p. sal. + sal., IP 0.3 ml saline/100 g body weight (1st injection) followed by 0.3 ml saline/100 g body weight (30 min later, 2nd injection); KEKS, ketone ester/KE + ketone salt/KS; LPS, lipopolysaccharide; PT days, post-treatment control days.

Rats were assigned into 6 groups (group 1–group 6; Figure 1). After the 2 week recovery period, animals were handled daily and were connected to the biological amplifier for 5 days for the adaptation of rats to the experimental procedures (e.g., EEG recording). Moreover, all rats (group 1–group 6) were fed paste-like standard rodent chow (standard rodent chow was mixed with water and 1% saccharine was added, without KEKS) between the 1st and 5th days of the experiments (adaptation to EEG recording and food). Then, to establish the averaged control SWD number (group 1–group 6) and average SWD time, discharge frequency within SWDs and time of sleep-waking stages (group 1), rats were fed by paste-like normal rat food (without KEKS) furthermore on 5 consecutive days between 6th and 10th days of the experiments, and EEG was recorded (5 day control period, pre-treatment control days) (Figure 1). In addition, except for the animals of group 1, all rats (group 2–group 6) were injected IP with 0.3 ml saline/100 g body weight (1st injection) followed by the same injection (0.3 ml saline/100 g body weight, 30 min later; 2nd injection) at the 5 day control period.

#### Treatments by Ketone Supplements, Indomethacin, and Lipopolysaccharide

In group 1 (*n* = 8), after the 5 day control period, animals were fed with KEKS food for 10 consecutive days (between the 11th and 20th days of the experiment) (Figure 1). Finally, the 10-day KEKS treatments was followed by two post-treatment control days (PT days; rats were fed by paste-like normal rat food without KEKS) to investigate putative sustained effect of KEKS on SWD number (on 21st and 22nd day of the experiment). EEGs were recorded every day.

After 5 day control periods, animals in groups 2–4 were treated by two IP injections (1st injection was followed by 2nd injection 30 min later) on the 11th day of the experiments (Figure 1): indomethacin (10 mg/kg) in 0.3 ml 5% ethanol solution/100 g body weight (1st injection) and 0.3 ml saline/100 g body weight (2nd injection; *n* = 8; group 2); 0.3 ml 5% ethanol solution/100 g body weight (1st injection) and LPS (50 μg/kg) in 0.3 ml saline/100 g body weight (2nd injection; *n* = 8; group 3); combined injection of indomethacin with LPS: indomethacin (10 mg/kg) in 0.3 ml 5% ethanol solution/100 g body weight (1st injection) and LPS (50 μg/kg) in 0.3 ml saline/100 g body weight (2nd injection; *n* = 8; group 4). Similar to group 1, EEGs were recorded every day.

After 5 day control periods, animals of groups 5 and 6 were fed with KEKS food for 10 consecutive days between the 11th and 20th days of the experiment. In addition, these animals received IP 0.3 ml saline/100 g body weight (1st injection) followed by the same injection (0.3 ml saline/100 g body weight, 30 min later; 2nd injection) between the 11th and 18th days and on the 20th day of the experiment (Figure 1). On the 19th day of the experiment, when KEKS treatment alone (group 1) significantly changed the SWD number, treatments were as follows in groups 5 and 6: 1st IP injection was 5% ethanol solution and the 2nd injection (saline) contained LPS (50 μg/kg; *n* = 8; group 5). In group 6, the 1st IP injection (5% ethanol solution) contained indomethacin (10 mg/kg) whereas the 2nd injection (saline) contained LPS (50 μg/kg; *n* = 8) on the 19th day of experiment (Figure 1). EEGs were recorded every day.

#### Measuring of Blood Glucose and βHB Levels as Well as Body Weight

Blood βHB (mmol/L) and glucose (mg/dl) levels were measured from blood taken from the tail vein with a glucose and ketone monitoring system (Precision Xtra™, Abbott Laboratories, Abbott Park, IL, USA) (Ari et al., 2016; Kovács et al., 2017), which only measures blood levels of D-βHB. Thus, total blood ketone levels (D-βHB + L-βHB + acetoacetate + acetone) would be higher than we measured. To investigate the effect of KEKS on blood glucose and βHB levels, we measured them on the last (5th) control day (control), on the days of the 1st and the 10th day of KEKS administration and on the 2nd PT day (on the 10th, 11th, 20th, and 22nd days of the experiment, respectively; group 1) (Figure 1).

The body weights of the rats were also measured before KEKS administration started (5th control day: control), after the last (10th) KEKS day and on the 2nd PT day (on the 10th, 20th, and 22nd day of the experiment, respectively; group 1).

### Evaluation of EEG Recordings and Statistical Analysis

Handling may evoke stress-induced changes in the behavior of WAG/Rij rats for about 30 min, which can modify the SWD number (Coenen and Van Luijtelaar, 2003; Kovács et al., 2006, 2014a). Thus, evaluation of the SWD number, average SWD time, discharge frequency within SWDs and sleep-waking stages were carried out between 30 and 150 min of recording. However, normal behavior and typical SWDs were detected in all animals 30 min after the connection of rats to the biological amplifier similarly to our previous study (Kovács et al., 2017). EEG recordings were split into 60 min sections and were evaluated separately (Kovács et al., 2006). Manual separation of SWDs from the EEG was carried out based on their features: typical SWDs contain a train of asymmetric spikes and slow waves starting and ending with sharp spikes, which SWDs characterized by 7–11 Hz discharge frequency within SWDs and 1–30 s duration (Coenen and Van Luijtelaar, 2003). SWDs were checked by FFT (Fast Fourier Transform) analysis.

It has been demonstrated previously that gavage of ketone supplements (e.g., KE) and ketone supplement-evoked ketosis had no effects on sleep-waking stages, average SWD time and discharge frequency within SWDs (Kovács et al., 2017). However, as we had no data on putative effects of KEKS food on sleep-waking stages, average SWD time and discharge frequency within SWDs, and also because changes in time of sleep-waking stages may alter SWD number in WAG/Rij rats (Coenen and Van Luijtelaar, 2003), we investigated the effect of KEKS food (after 9th day of KEKS administration; 19th day of experiments; group 1) on SWD number, average time of SWDs, discharge frequency within SWDs and sleep-waking stages between 30 and 90 min. Evaluation of sleep-waking stages was performed offline by visual evaluation of the raw EEG. We distinguished wakefulness (passive and active wake), slow wave sleep (SWS; light SWS and deep SWS) and rapid eye movement (REM) sleep in 60 min epochs according to Kovács et al. (2006). The wakefulness stage shows predominantly beta (20–40 Hz) and theta (6–8 Hz) activity (passive/active wake: without/with high slow waves of motor artifacts); light SWS was characterized by sleep spindles (10–16 Hz), theta waves and some slow waves (2–4 Hz); the disappearance of sleep spindles and an increasing ratio of high slow delta waves (0.5–4 Hz) were demonstrated under deep SWS; and REM sleep was characterized by continuous theta activity without any motor artifacts (Kovács et al., 2006).

All results were expressed as means ± standard error of the mean (S.E.M.). In relation to SWD number, average SWD time, discharge frequency within SWDs and sleep-waking stages the pre-treatment control values were the grand average calculated from the results of 5 control days (5 day control period). In case of blood level of glucose, βHB and body weight, the results were calculated from the values measured on the last (5th) control day. Data analysis was performed using GraphPad PRISM version 6.0a. Significance was determined by One- or Two-way analysis of variance (ANOVA) with Tukey's multiple comparisons test and Sidak's multiple comparisons test as was described previously (Ari et al., 2016). Results were considered significant when *p* < 0.05.

## Results

### Effect of the KEKS Treatment on Absence Epileptic Activity, Sleep-Waking Stages, Blood βHB, and Glucose Levels as Well as Body Weight

**Figure 2:**
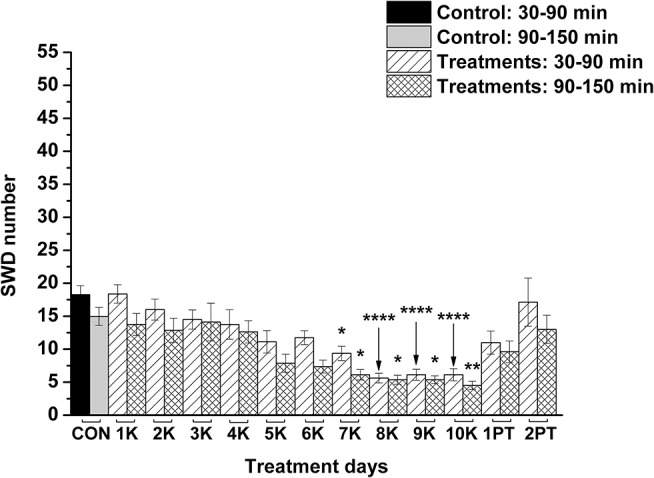
Effect of KEKS food (20% KEKS and 1% saccharin in standard rodent chow; group 1) administration on SWD number. 1K, 1st KEKS food administration; 2K, 2nd KEKS food administration and so on; 1PT, 1st post-treatment control day; 2PT, 2nd post-treatment control day; CON, control; SWD, spike-wave discharge. ^*^p < 0.05, ^**^p < 0.01, and ^****^p < 0.0001.

KEKS food significantly decreased the SWD number between the 7th and 10th days of the treatment from 30 to 150 min (group 1; Figure 2; Table 1), compared to control levels. Moreover, SWD numbers returned to near the control levels on the 2nd PT day (Figure 2; Table 1). In relation to discharge frequency within SWDs, we did not find significant changes on the 9th KEKS treatment day between 30 and 90 min, compared to the control (group 1; Table 2). Average SWD duration was similar to the control on the 9th day of KEKS treatment between 30 and 90 min (group 1; Table 2). Because average SWD duration did not change after treatment by KEKS, but SWD number decreased (Figure 2; group 1), changes in the total time of SWDs were similar to the change in SWD number. Indeed, on the 9th day of KEKS treatment, the total time of SWDs decreased between 30 and 90 min compared to the control (Table 2).

**Table 1:** Effect of KEKS food (20% KEKS and 1% saccharin in standard rodent chow; Figure 2, group 1) on SWD number.

**Treatments (Figure 2; group 1)**	**SWD number (mean ± S.E.M.; significance level/*p*-value)**	
	**30–90 min**	**90–150 min**
Control (CON)	18.3 ± 1.352	14.9 ± 1.358
1st KEKS treatment (1K)	18.4 ± 1.388 –/>0.9999	13.8 ± 1.666 –/>0.9999
2nd KEKS treatment (2K)	16.0 ± 1.592 –/>0.9999	12.9 ± 1.817 –/>0.9999
3rd KEKS treatment (3K)	14.5 ± 1.452 –/0.9956	14.1 ± 2.850 –/>0.9999
4th KEKS treatment (4K)	13.8 ± 2.242 –/0.9586	12.6 ± 1.689 –/>0.9999
5th KEKS treatment (5K)	11.1 ± 1.705 –/0.2504	7.9 ± 1.381 –/0.2692
6th KEKS treatment (6K)	11.8 ± 1.048 –/0.4306	7.4 ± 0.981 –/0.1607
7th KEKS treatment (7K)	9.4 ± 1.085 ^*^/0.0288	6.1 ± 0.789 ^*^/0.0321
8th KEKS treatment (8K)	5.6 ± 0.730 ^****^/<0.0001	5.4 ± 0.679 ^*^/0.0102
9th KEKS treatment (9K)	6.1 ± 0.833 ^****^/<0.0001	5.4 ± 0.596 ^*^/0.0102
10th KEKS treatment (10K)	6.1 ± 0.915 ^****^/<0.0001	4.5 ± 0.627 ^**^/0.0023
1st Post-treatment day (1PT)	11.0 ± 1.753 –/0.2211	9.6 ± 1.625 –/0.8168
2nd Post-treatment day (2PT)	17.1 ± 3.652 –/>0.9999	13.0 ± 2.129 –/>0.9999

*1st KEKS treatment, 1st day of KEKS food application; SWD, spike-wave discharge*.

* *p < 0.05*,

** p < 0.01 and

**** *p < 0.0001*.

**Table 2:** Effect of KEKS food (20% KEKS and 1% saccharin in standard rodent chow; group 1) on discharge frequency within SWDs, average SWD duration and total time of SWDs on 9th KEKS treatment day between 30 and 90 min.

**Treatments (group 1)**	**Discharge frequency within SWDs (Hz ± S.E.M.; significance level/*p*-value)**	**Average SWD duration (sec ± S.E.M.; significance level/*p*-value)**	**Total time of SWDs (sec ± S.E.M.; significance level/*p*-value)**
Control	7.6 ± 0.101	6.1 ± 0.334	111.8 ± 3.327
KEKS	7.5 ± 0.092 –/0.3655	5.8 ± 0.354 –/0.6550	35.6 ± 1.525 ^****^/<0.0001

*SWD, spike-wave discharge*.

**** *p < 0.0001*.

The total time of sleep-waking stages did not change on the 9th day of KEKS food administration, compared to the control (group 1; Table 3).

**Table 3:** Effect of KEKS food (20% KEKS and 1% saccharin in standard rodent chow; group 1) on total time of sleep-waking stages on 9th KEKS treatment day between 30 and 90 min.

**Treatments (group 1)**	**Active wake (sec; mean ± S.E.M.; significance level/*p*-value)**	**Passive wake (sec; mean ± S.E.M.; significance level/*p*-value)**	**Light SWS (sec; mean ± S.E.M.; significance level/*p*-value)**	**Deep SWS (sec; mean ± S.E.M.; significance level/*p*-value)**	**REM (sec; mean ± S.E.M.; significance level/*p*-value)**
Control	678.5 ± 39.002	667.9 ± 14.909	953.2 ± 27.649	1,081.8 ± 43.387	106.9 ± 6.403
KEKS	682.6 ± 38.976 –/0.9407	681.9 ± 14.226 –/0.5046	942.1 ± 16.312 –/0.7358	1145.1 ± 48.361 –/0.3463	112.5 ± 3.524 –/0.4563

*REM, rapid eye movement sleep; SWS, slow wave sleep*.

Blood βHB level was significantly increased after the 1st and 10th KEKS treatment compared to control levels, whereas βHB level returned to the baseline (control) levels on the 2nd PT day (group 1; Table 4). Glucose level was unchanged on the days tested (group 1; Table 4).

**Table 4:** Effect of KEKS food (20% KEKS and 1% saccharin in standard rodent chow; group 1) on blood βHB and glucose levels.

**Treatments (group 1)**	**Blood βHB and glucose levels (mean ± S.E.M.; significance level/*p*-value)**	
	**βHB (mmol/l)**	**Glucose (mg/dl)**
Control (CON)	0.83 ± 0.025	70.88 ± 2.022
1st KEKS treatment (1K)	1.25 ± 0.059 ^***^/0.0007	66.75 ± 1.924 –/0.3571
10th KEKS treatment (10K)	1.35 ± 0.109 ^****^/<0.0001	73.88 ± 1.407 –/0.6224
2nd post-treatment day (2PT)	0.80 ± 0.046 –/0.9935	76.13 ± 1.552 –/0.1692

*1st KEKS treatment, 1st day of KEKS food administration; βHB, beta-hydroxybutyrate*.

*** p < 0.001 and

**** *p < 0.0001*.

After KEKS treatment, no significant change in body weight was detected compared to control levels (group 1) (body weight, g ± S.E.M.; on the control day/10th KEKS day: 331.8 ± 5.694/324.8 ± 5.192, *p* = 0.823; on control day/2nd PT day: 331.8 ± 5.694/329.5 ± 5.096, *p* = 0.964). Although food intake was not monitored in this study, the unchanged body weight suggests that KEKS treatment did not exert its effect on epileptic activity by means of insufficient food/energy intake and calorie restriction.

### Indomethacin- and Lipopolysaccharide-Evoked Influences on the Absence Epileptic Activity

**Figure 3:**
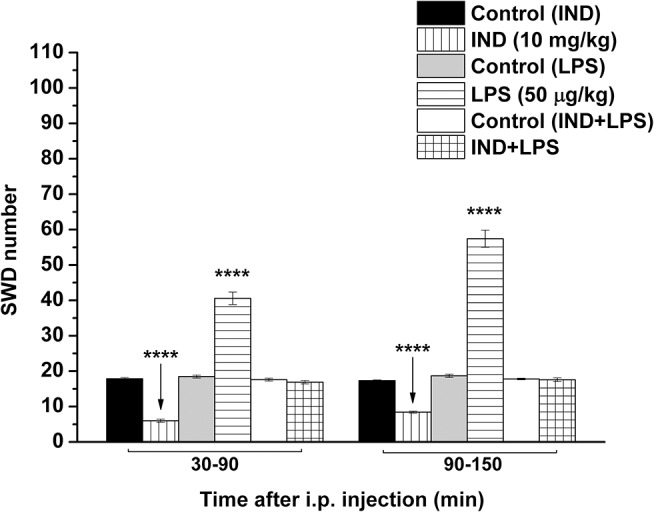
Effect of IP indomethacin (IND: 10 mg/kg), IP lipopolysaccharide (LPS: 50 μg/kg), and combined administration of IP indomethacin (10 mg/kg) and IP LPS (50 μg/kg; IND+LPS) on SWD number (groups 2, 3, and 4, respectively). IND, indomethacin; i.p., intraperitoneal; LPS, lipopolysaccharide; SWD, spike-wave discharge. ^****^p < 0.0001 level of significance.

As we demonstrated previously (Kovács et al., 2006, 2014a), IP indomethacin (10 mg/kg) alone significantly decreased the SWD number between 30 and 150 min compared to the control (group 2; Figure 3; Table 5). Moreover, IP LPS (50 μg/kg) alone significantly increased the SWD number between 30 and 150 min compared to the control (group 3; Figure 3; Table 5). Combined IP administration of indomethacin (10 mg/kg) with LPS (50 μg/kg) abolished the effect of LPS alone on SWD number (group 4; Figure 3; Table 5).

**Table 5:** Effect of IP indomethacin and lipopolysaccharide alone, as well as their combined administration on SWD number (Figure 3, group 2–4).

**Treatments (Figure 3; groups 2–4)**	**SWD number (mean ± S.E.M.; significance level/*p*-value)**	
	**30–90 min**	**90–150 min**
Control	17.9 ± 0.306	17.3 ± 0.189
Indomethacin	6.0 ± 0.436 ^****^/<0.0001	8.4 ± 0.297 ^****^/<0.0001
Control	18.5 ± 0.413	18.7 ± 0.457
LPS	40.6 ± 1.757 ^****^/<0.0001	57.4 ± 2.419 ^****^/<0.0001
Control	17.6 ± 0.408	17.8 ± 0.233
Indomethacin + LPS	16.9 ± 0.488 –/0.6321	17.6 ± 0.528 –/0.9773

*LPS, lipopolysaccharide; SWD, spike-wave discharge*.

**** *p < 0.0001*.

### Effect of the KEKS Treatment on Lipopolysaccharide-Evoked Changes in Absence Epileptic Activity

**Figure 4:**
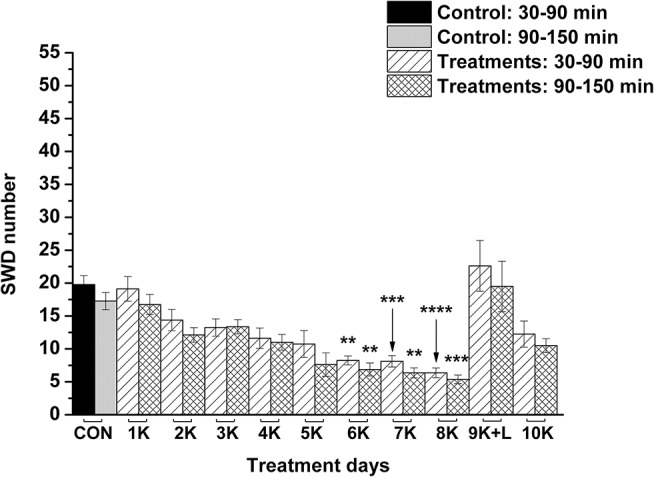
The effect of combined administration of KEKS food (20% KEKS and 1% saccharin in standard rodent chow) with IP LPS (50 μg/kg) on SWD number on the day of 9th KEKS food administration (9K+L; group 5). 1K, 1st KEKS food administration; 2K, 2nd KEKS food administration and so on; 9K+L, combined administration of KEKS food (K) with i.p. LPS (L) on the day of 9th KEKS food administration; CON, control; SWD, spike-wave discharge. ^**^p < 0.01, ^***^p < 0.001, and ^****^p < 0.0001.

**Figure 5:**
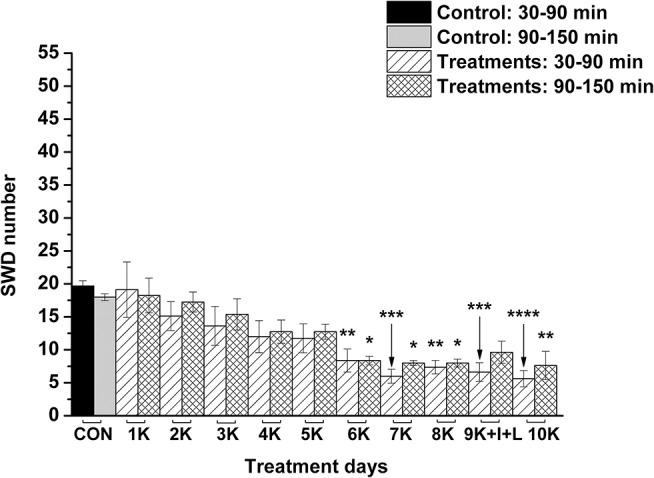
The effect of combined administration of KEKS food (20% KEKS and 1% saccharin in standard rodent chow) with IP indomethacin plus IP LPS (10 mg/kg + 50 μg/kg, respectively) on SWD number on the day of 9th KEKS food administration (9K+I+L; group 6). 1K, 1st KEKS food administration; 2K, 2nd KEKS food administration and so on; 9K+I+L, combined administration of KEKS food (K) with i.p. indomethacin (I) and i.p. LPS (L) on the day of 9th KEKS food administration; CON, control; SWD, spike-wave discharge. ^*^p < 0.05, ^**^p < 0.01, ^***^p < 0.001, and ^****^p < 0.0001.

Nevertheless, KEKS treatment decreased the SWD number between the 6th and 8th days of the treatment from 30 to 150 min (group 5 and 6; Figures 4, 5; Tables 6, 7), and administration of KEKS food abolished the aggravating effect of IP LPS (50 μg/kg) on SWD number on the 9th KEKS day (group 5; Figure 4; Table 6). As Figure 5 shows, IP indomethacin (10 mg/kg) enhanced the alleviating effect of KEKS treatment on the IP LPS-evoked increase in SWD number on the 9th KEKS day; after combined injection of indomethacin with LPS, a decrease in SWD number was demonstrated between 30 and 150 min (Figure 5; Table 7), with the influence being significant between 30 and 90 min. One day after the 9th KEKS day (KEKS food administration with IP LPS injection; group 5), the SWD number was not different, compared to the control on the 10th KEKS day (Figure 4; Table 6). Nevertheless, one day after the 9th KEKS food administration, which was combined with an IP injection of indomethacin and LPS (group 6), the SWD number significantly decreased compared to the control on the 10th KEKS day (Figure 5; Table 7).

**Table 6:** Effect of KEKS food on IP lipopolysaccharide-evoked changes in SWD number on the 9th KEKS day (9K+L; Figure 4, group 5).

**Treatments (Figure 4; group 5)**	**SWD number (mean ± S.E.M.; significance level/*p*-value)**	
	**30–90 min**	**90–150 min**
Control (CON)	19.8 ± 1.366	17.3 ± 1.301
1st KEKS treatment (1K)	19.1 ± 1.875 –/>0.9999	16.8 ± 1.532 –/>0.9999
2nd KEKS treatment (2K)	14.4 ± 1.625 –/0.8297	12.1 ± 1.109 –/0.8764
3rd KEKS treatment (3K)	13.3 ± 1.306 –/0.5073	13.4 ± 1.085 –/0.9923
4th KEKS treatment (4K)	11.6 ± 1.558 –/0.1289	11.0 ± 1.195 –/0.5773
5th KEKS treatment (5K)	10.8 ± 2.024 –/0.2122	7.6 ± 1.792 –/0.1358
6th KEKS treatment (6K)	8.3 ± 0.675 ^**^/0.0084	6.9 ± 0.989 ^**^/0.0067
7th KEKS treatment (7K)	8.1 ± 0.875 ^***^/0.0009	6.4 ± 0.754 ^**^/0.0031
8th KEKS treatment (8K)	6.4 ± 0.730 ^****^/<0.0001	5.4 ± 0.653 ^***^/0.0006
9th KEKS + LPS treatment (9K+L)	22.6 ± 3.868 –/>0.9999	19.5 ± 3.827 –/>0.9999
10th KEKS treatment (10K)	12.3 ± 1.989 –/0.2380	10.5 ± 1.052 –/0.4243

*1st KEKS treatment, 1st day of KEKS food administration; LPS, lipopolysaccharide; SWD, spike-wave discharge*.

** *p < 0.01*,

*** p < 0.001, and

**** *p < 0.0001*.

**Table 7:** Effect of KEKS food on IP indomethacin plus lipopolysaccharide-evoked changes in SWD number on the 9th KEKS day (9K+I+L; Figure 5, group 6).

**Treatments (Figure 5; group 6)**	**SWD number (mean ± S.E.M.; significance level/*p*-value)**	
	**30–90 min**	**90–150 min**
Control (CON)	19.7 ± 0.783	17.9 ± 0.508
1st KEKS treatment (1K)	19.1 ± 4.223 –/>0.9999	18.3 ± 2.631 –/>0.9999
2nd KEKS treatment (2K)	15.1 ± 2.191 –/0.9819	17.3 ± 1.521 –/>0.9999
3rd KEKS treatment (3K)	13.6 ± 2.964 –/0.7755	15.4 ± 2.375 –/>0.9999
4th KEKS treatment (4K)	12.0 ± 2.435 –/0.3315	12.8 ± 1.770 –/0.9261
5th KEKS treatment (5K)	11.8 ± 2.194 –/0.3626	12.7 ± 1.129 –/0.9261
6th KEKS treatment (6K)	8.4 ± 1.752 ^**^/0.0059	8.4 ± 0.653 ^*^/0.0396
7th KEKS treatment (7K)	6.0 ± 1.069 ^***^/0.0001	8.0 ± 0.378 ^*^/0.0335
8th KEKS treatment (8K)	7.4 ± 1.017 ^**^/0.0014	8.1 ± 0.598 ^*^/0.0335
9th KEKS + indomethacin + LPS treatment (9K+I+L)	6.6 ± 1.426 ^***^/0.0004	9.6 ± 1.700 –/0.0794
10th KEKS treatment (10K)	5.6 ± 1.238 ^****^/<0.0001	7.6 ± 2.138 ^**^/0.0077

*1st KEKS treatment, 1st day of KEKS food administration; LPS, lipopolysaccharide; SWD, spike-wave discharge*.

* *p < 0.05*,

** *p < 0.01*,

*** p < 0.001, and

**** *p < 0.0001*.

## Discussion

Our results provide new evidence that exogenous ketone (KEKS)-supplemented food could decrease the LPS-evoked increase in SWD number. Moreover, we further confirmed our previous results that IP indomethacin alone decreases SWD number and IP LPS alone increases SWD number, whereas combined administration of indomethacin with LPS abolishes the LPS-generated increase in SWD number (Kovács et al., 2006, 2011, 2014a). However, similar to our previous results, when oral gavage of ketone supplements (e.g., KE) was administered to WAG/Rij rats for 7 days (Kovács et al., 2017), 10 days of administering KEKS food also had no significant effects on discharge frequency within SWDs, total time of sleep-waking stages, blood glucose levels or body weight of WAG/Rij animals in the recent study.

It was demonstrated previously that administration of exogenous ketone supplements is a well-tolerated, safe and efficient method to evoke therapeutic ketosis in both animals and humans (Newport et al., 2015; Kesl et al., 2016; Stubbs et al., 2017). After consumption, digestion of exogenous ketone supplements such as KEKS (or KEKS supplemented food) in the small intestine liberates ketone bodies (e.g., βHB and AcAc) (Brunengraber, 1997), which can be transported to the systemic bloodstream and evoke rapidly and then maintain mild therapeutic ketosis (Ari et al., 2016; Kesl et al., 2016; Stubbs et al., 2017). Subsequently, ketone bodies can be transported to the mitochondria of brain cells. In the mitochondria, ketone bodies are converted back to acetyl-CoA, a molecule which enters the Krebs cycle and is then used as an energy source under different circumstances when glucose supply is insufficient, alleviating cell energy metabolism (Newman and Verdin, 2014; Achanta and Rae, 2017). It was also demonstrated that an exogenous ketone supplement-evoked increase in ketone body/βHB concentration may evoke beneficial effects on different CNS diseases in animal models and/or humans (D'Agostino et al., 2013; Hashim and VanItallie, 2014; Newport et al., 2015; Ari et al., 2016), such as epilepsy (Kovács et al., 2017; Simeone et al., 2018). In relation to the mechanism of action on absence epileptic activity, an increased level of ketone bodies/βHB is able to inhibit glycolysis. Subsequently, this process results in a decrease in cytosolic ATP level near the plasma membrane, which enhances the activity of ATP-sensitive potassium (K_ATP_) channels hyperpolarizing neuronal membranes (Achanta and Rae, 2017) and decreasing seizure activity (Boison and Steinhäuser, 2018). Moreover, βHB may modulate release of neurotransmitters, among others, by inhibition of histone deacetylase (Sleiman et al., 2016). Indeed, ketosis/βHB enhances the GABAergic effects (e.g., via increased levels/activity of GABA and GABA_A_ receptors), decreases extracellular glutamate release and increases adenosine level (McNally and Hartman, 2012; Sharma et al., 2015; Achanta and Rae, 2017), which exerts influence by effectively modulating absence epileptic activity (Peeters et al., 1989; D'Alimonte et al., 2009; Kovács et al., 2015a). As we demonstrated previously, after oral gavage of ketone supplements, such as KE, absence epileptic activity was decreased in correlation with the increase in βHB levels in WAG/Rij rats. This likely happens via inhibitory A1 type of adenosine receptors (A_1_Rs) (Kovács et al., 2017), because activation of both the A2A type of adenosine receptors (A_2A_Rs) and GABA_A_ receptors evoked an increase in the SWD number in WAG/Rij rats (D'Alimonte et al., 2009; Kovács et al., 2015a; Lakatos et al., 2016). Activation of A_1_Rs may decrease neuronal activity by hyperpolarization of neuronal membranes via synaptic inhibition (e.g., by decreased Ca^2+^ influx) and by modulation of neurotransmitter release (e.g., by decreased glutamate release) (Ciruela et al., 2006; Kovács et al., 2014b). It has been demonstrated that activation of A_1_Rs causes hyperpolarization with opening potassium channels (Haas and Greene, 1984) and that a ketogenic diet (ketones) suppresses hyperexcitability of neurons by opening K_ATP_ channels via activation of A_1_Rs (Kawamura et al., 2014). An exogenous ketone supplement-evoked increase in βHB concentration may increase the activity of mitochondrial synthesis of ATP in brain cells (Achanta and Rae, 2017; Simeone et al., 2018). ATP may release from cells and metabolize to adenosine by ectonucleotidases (Kovács et al., 2013), an effect (increased extracellular adenosine level) which may evoke opening of K_ATP_ channels via A_1_Rs and, consequently, may generate hyperpolarization of the neuronal membrane and decrease neuronal activity (Andoh et al., 2006; Achanta and Rae, 2017; Simeone et al., 2018). All these effects may decrease excess hyperexcitability in the somatosensory cortex (cortical focus of absence epilepsy genesis) (Coenen and Van Luijtelaar, 2003), decreasing SWD number in WAG/Rij rats. Our results suggest that KEKS food-evoked ketosis may also decrease SWD number (Figure 2; Table 1) *via* increased adenosine levels and by activation of A_1_Rs as well as K_ATP_ channels.

It is widely recognized that glial cells have a leading role both in the genesis and modulation of epileptic activity, for example, by modification of inflammatory processes (Boison and Steinhäuser, 2018; Hiragi et al., 2018). Indeed, it has been demonstrated that IP injection of LPS evokes an immune response in the brain by means of TLR4, glycosylphosphatidylinositol-anchored glycoprotein CD14 and accessory protein MD-2 (Vezzani and Granata, 2005; Vezzani et al., 2011). LPS generates the release of proinflammatory cytokines such as IL-1β and tumor necrosis factor-α (TNF-α) from activated glial cells by means of the TLR4 pathway (Mlodzikowska-Albrecht et al., 2007; Vezzani et al., 2011). IL-1β and TNF-α may modulate excitatory and inhibitory processes via increased glutamatergic and decreased GABAergic neurotransmission (Vezzani and Granata, 2005; Mlodzikowska-Albrecht et al., 2007; Vezzani et al., 2008, 2011) and may generate an imbalance between excitatory/inhibitory processes in the brain; LPS, IL-1β and prostanoids (e.g., PGE_2_) may evoke neuronal hyperexcitability and rapid excitation in the cortex via the TLR4/IL-1 receptor (IL-1R) signaling pathway (Wang and White, 1999; Maroso et al., 2011). As SWDs arise from the hyperexcitable focal cortical zone in WAG/Rij rats, these LPS-induced inflammatory processes above aggravate absence epileptic activity/SWD number via an increase in excitation in the thalamo-cortical/cortico-thalamic circuitry (Coenen and Van Luijtelaar, 2003; Kovács et al., 2006, 2014a). Moreover, enhanced activity of astrocytes by inflammatory processes may evoke increased activity of the adenosine-metabolizing enzyme adenosine kinase (Boison and Steinhäuser, 2018). Under these circumstances, adenosine kinase intensifies the metabolism of adenosine to adenosine monophosphate (Kovács et al., 2014b) and, as a result, it decreases adenosine levels and may increase not only neuronal excitability, but also the SWD number via decreased activity of inhibitory A_1_Rs.

NOD-like receptor pyrin domain 3 (NLRP3) inflammasome is a multiprotein complex containing NOD-like receptor (NLRP3), an adaptor protein (ASC: apoptosis-associated speck-like protein containing a caspase recruitment domain) and a cysteine protease (caspase-1) (de Rivero Vaccari et al., 2014; Levy et al., 2015), which has a role in the pathophysiology of several CNS diseases, such as epilepsy (Edye et al., 2014; Simeone et al., 2018). The NLRP3 inflammasome controls caspase-1 activity and release of proinflammatory cytokines (e.g., IL-1β). Several factors, such as reactive oxygen species (ROS) and LPS (e.g., activation of TLR4-induced nuclear factor-κB/NF-κB pathway) have a role in the activation of NLRP3 inflammasome: first, translocation of gene expression regulator NF-κB generates upregulation of NLRP3 and pro-IL-1β expression. Subsequently, activation of inflammasome evokes activation of caspase-1-dependent cleavage of pro-IL-1β to its active form (IL-1β) for secretion (Levy et al., 2015; Patel et al., 2017). It has been demonstrated that βHB can exert its antiinflammatory effects by inhibition of NLRP3 and NLRP3 inflammasome-mediated cytokine production/inflammatory processes, suggesting a strong interaction between stimulation of the immune system and metabolic processes. For example, βHB decreased the expression/level of NLRP3, ASC, caspase-1 and IL-1β (Bae et al., 2016), attenuated the release of IL-1β in human monocytes (Youm et al., 2015), and mitigated stress-induced increases in TNF-α and IL-1β in the hippocampus (Yamanashi et al., 2017). Moreover, βHB attenuates the LPS-evoked increase in IL-1β and TNF-α level, as well as LPS-generated increases in COX-2, IL-1β, and TNF-α mRNA expression in BV-2 cells, likely via inhibition of NF-κB signaling (Fu et al., 2014). It was also demonstrated that βHB may decrease inflammatory processes (e.g., expression of COX and IL-1β) via its G-protein-coupled receptor 109A (GPR109A or hydroxyl-carboxylic acid receptor 2/HCA2), which evoked inhibitory influence on the NF-κB signaling pathway in microglial cells (Fu et al., 2015; Graff et al., 2016). Thus, it is possible that KEKS food-evoked ketosis/increased βHB level may suppress the LPS-generated inflammatory processes and, as a consequence, may evoke antiepileptic effects in WAG/Rij rats via inhibition of the TLR4/IL-1R/NF-κB signaling pathway and decreasing the release of proinflammatory cytokines/enzymes (e.g., IL-1β and COX-2). This hypothesis is supported by our results, in which IP indomethacin enhanced the antiepileptic influence of KEKS food (Figures 4, 5), likely via inhibition of the COX-2/PGE2 system. Moreover, it has been demonstrated that βHB may decrease production of ROS (Maalouf et al., 2007). As ROS may trigger/enhance inflammasome activation and release of proinflammatory cytokines (Patel et al., 2017), it is also possible that KEKS food-evoked increases in βHB level may decrease activation of NLRP3 inflammasome and the subsequent increase in IL-1β level via modulation of ROS production (Choi and Nakahira, 2011; Bae et al., 2016), effects which may decrease the SWD number. In addition, KEKS food-evoked ketosis may also generate anti-inflammatory/antiepileptic effects via increased extracellular adenosine level: for example, adenosine may decrease LPS-induced cytokine production of microglial cells via A_2A_Rs (Van der Putten et al., 2009). Nevertheless, adenosine may attenuate the deleterious influence of ROS on brain cells via A_1_Rs (Almeida et al., 2003).

It was suggested previously that inflammatory processes and proinflammatory cytokines may have a role in the genesis/modulation of spontaneous absence epileptic activity in WAG/Rij rats (Kovács et al., 2006, 2011; Van Luijtelaar et al., 2012; Russo et al., 2014). For example, antiinflammatory drugs, such as indomethacin and a selective COX-2 inhibitor etoricoxib, may decrease not only the LPS-induced increase in SWD number (indomethacin) but also spontaneous absence seizures (indomethacin and etoricoxib) (Kovács et al., 2006, 2011, 2014a; Citraro et al., 2015), whereas etoricoxib evoked long-lasting antiepileptogenic effects likely by inhibition of background inflammatory processes (Citraro et al., 2015). Based on these and our recent results, it is possible that exogenous ketone supplements exert their alleviating effect on both spontaneous absence epileptic activity (Kovács et al., 2017) (Figure 2) and LPS-induced increases in SWD number (Figure 4) via inhibition of inflammatory processes. Moreover, our results further supported the hypothesis that inflammation and changes in proinflammatory cytokine/enzyme levels could play a role in the appearance/modulation of absence epileptic activity in WAG/Rij rats, and likely other forms of seizures. For example, it is also well established that pro-inflammatory mediators evoke epileptogenic and ictogenic properties following traumatic brain injury (Webster et al., 2017), and this suggests that the therapeutic potential of ketone supplementation to target these inflammatory pathways may be an effective mitigation strategy for post-traumatic epilepsy, especially with penetrating brain injuries where neuroinflammation and seizure occurrence is very high.

In conclusion, KEKS food-evoked ketosis/βHB may decrease the neuronal activity/cortical excitability, which could decrease spontaneous and LPS-generated increases in absence epileptic activity. βHB may exert its antiepileptic effect via multiple processes by changes in metabolic pathways (e.g., modulating ATP level and activity of K_ATP_ channels), neurotransmitter systems (e.g., increasing adenosine level/release and activity of A_1_Rs) and the function/activity of the TLR4/IL-1R/NF-κB/COX pathways (e.g., inhibiting proinflammatory cytokine production and release). Our results suggest that ketogenic foods containing exogenous ketone supplements such as KEKS food not only evoke ketosis but, as a consequence, also alleviate the inflammation-evoked processes in the CNS. Thus, theoretically, administration of exogenous ketogenic supplements (ketogenic foods) may be a promising therapeutic tool/metabolic therapy in the treatment of epilepsy and other inflammation-generated neurodegenerative diseases. Therefore, further studies, such as detailed investigation of influence of LPS administration in combination with different ketone supplemented foods on neuronal and glial cells are needed to reveal the exact mechanism of action of exogenous ketone supplementation-induced ketosis on LPS/inflammation-evoked changes in the CNS.

## Data Availability

The datasets generated for this study are available on request to the corresponding author.

## Author Contributions

ZK: conception and design of experiments, data collection, interpretation of data, and writing manuscript. DPD: revising manuscript. DMD: revising manuscript. CA: data analysis and revising manuscript.

### Conflict of Interest Statement

International Patent # PCT/US2014/031237, University of South Florida, DPD, S. Kesl, and P. Arnold, Compositions and Methods for Producing Elevated and Sustained Ketosis. Non-provisional patents: #62289749, University of South Florida, CA and DPD, Exogenous ketone supplements for reducing anxiety-related behavior; CA, Arnold P, and DPD Technology Title: Elevated Blood Ketone Levels by Ketogenic Diet or Exogenous Ketone Supplements Induced Increased Latency of Anesthetic Induction USF Ref. No. 16A018PR; CA, Arnold P, and DPD Technology Title: Exogenous Ketone Supplementation Improved Motor Function in Sprague-Dawley Rats. USF Ref. No: 16A019; CA, Arnold P, and DPD Technology Title: Lowering of Blood Glucose in Exercising and Non-Exercising Rats Following Administration of Exogenous Ketones and Ketone Formulas. USF Ref. No: 16A049; CA, Arnold P, and DPD Technology Title: Ketone Supplementation Elevates Blood Ketone Level and Improves Motor Function in GLUT1 Deficiency Syndrome Mice. USF Ref. No: 16B116 (provisional patent); CA, Arnold P, and DPD Technology Title: Neuroregeneration improved by ketone. USF Ref. No: 16B128 (provisional patent); CA, DPD, and Dean, J. B Technology Title: Delaying latency to seizure by combinations of ketone supplements. USF Ref. No: 16B138PR. DPD and AC are co-owners of the company Ketone Technologies LLC, providing scientific consulting and public speaking engagements about ketogenic therapies. The company obtained an option agreement from the University of South Florida on the non-provisional patent No. 62/310,302 Methods of increasing latency of anesthetic induction using ketone supplementation. These interests have been reviewed and managed by the University in accordance with its Institutional and Individual Conflict of Interest policies. The authors declare that Csilla Ari received funding from Quest Nutrition LLC. The funder was not involved in the study design or collection, analysis, or interpretation of the data.

## Abbreviations

A_1_R: adenosine A_1_ receptor
A_2A_R: adenosine A_2A_ receptor
ASC: apoptosis-associated speck-like protein containing a caspase recruitment domain
βHB: beta-hydroxybutyrate
CNS: central nervous system
COX: cyclooxygenase
EEG: electroencephalogram
IL-1β: interleukin-1β
i.p.: intraperitoneal
K_ATP_ channels: ATP-sensitive potassium channels
KE: ketone ester
KS: ketone salt
LPS: lipopolysaccharide
NF-κB: nuclear factor-κB
NLRP3: NOD-like receptor pyrin domain 3
PGE_2_: prostaglandin E_2_
PT day: post-treatment control experiment/day
REM sleep: rapid eye movement sleep
ROS: reactive oxygen species
S.E.M.: standard error of the mean
SWD: spike-wave discharge
SWS: slow wave sleep
TLR4: Toll-like receptor 4
TNF-α: tumor necrosis factor-α
WAG/Rij: Wistar Albino Glaxo/Rijswijk.
